# Manufacturing of 3D-Printed Hybrid Scaffolds with Polyelectrolyte Multilayer Coating in Static and Dynamic Culture Conditions

**DOI:** 10.3390/ma17122811

**Published:** 2024-06-08

**Authors:** Yanny Marliana Baba Ismail, Yvonne Reinwald, Ana Marina Ferreira, Oana Bretcanu, Kenneth Dalgarno, Alicia J. El Haj

**Affiliations:** 1 School of Materials and Mineral Resources Engineering, Universiti Sains Malaysia, Engineering Campus, Nibong Tebal 14300, Penang, Malaysia; 2 Guy Hilton Research Centre, Institute for Science and Technology in Medicine, Keele University, Thornburrow Drive, Hartshill, Stoke-on-Trent ST4 7QB, UK; 3 School of Mechanical and Systems Engineering, Newcastle University, Newcastle-upon-Tyne NE1 7RU, UK; 4 School of Science & Technology, Department of Engineering, Nottingham Trent University, Clifton Campus, Nottingham NG1 18NS, UK; 5 Medical Technology Innovation Facility, Nottingham Trent University, Clifton Campus, Nottingham NG1 18NS, UK; 6 Institute of Translational Medicine, Heritage Building (Old Queen Elizabeth Hospital), Mindelsohn Way, Birmingham B15 2TH, UK

**Keywords:** 3DP hybrid scaffolds, fused deposition modelling, polyelectrolyte multilayers, stem cells, rotating wall vessels, co-culture, pre-vascularisation

## Abstract

Three-dimensional printing (3DP) has emerged as a promising method for creating intricate scaffold designs. This study assessed three 3DP scaffold designs fabricated using biodegradable poly(lactic) acid (PLA) through fused deposition modelling (FDM): mesh, two channels (2C), and four channels (4C). To address the limitations of PLA, such as hydrophobic properties and poor cell attachment, a post-fabrication modification technique employing Polyelectrolyte Multilayers (PEMs) coating was implemented. The scaffolds underwent aminolysis followed by coating with SiCHA nanopowders dispersed in hyaluronic acid and collagen type I, and finally crosslinked the outermost coated layers with EDC/NHS solution to complete the hybrid scaffold production. The study employed rotating wall vessels (RWVs) to investigate how simulating microgravity affects cell proliferation and differentiation. Human mesenchymal stem cells (hMSCs) cultured on these scaffolds using proliferation medium (PM) and osteogenic media (OM), subjected to static (TCP) and dynamic (RWVs) conditions for 21 days, revealed superior performance of 4C hybrid scaffolds, particularly in OM. Compared to commercial hydroxyapatite scaffolds, these hybrid scaffolds demonstrated enhanced cell activity and survival. The pre-vascularisation concept on 4C hybrid scaffolds showed the proliferation of both HUVECs and hMSCs throughout the scaffolds, with a positive expression of osteogenic and angiogenic markers at the early stages.

## 1. Introduction

Tissue engineering (TE) is a multidisciplinary research field driven by the goal of restoring, replacing, or regenerating defective tissues [1]. In the context of bone tissue, the emergence of TE is due to the limited availability of suitable bone graft substitutes to treat patients with congenital defects, tumours, or non-union fractures. There are three main components in bone TE (BTE): a three-dimensional (3D) scaffold, a cell source, and chemical cues [2,3].

In scaffold-based BTE, a 3D biodegradable scaffold is designed to serve as a temporary substrate to allow cell growth and activity as well as encourage cells to synthesise their extracellular matrix (ECM) and other biological cues that could facilitate the formation of functional tissues/organs [2,4]. Precise control over the structural design of 3D porous scaffolds created through conventional methods remains limited. Scaffolds made using foam replication techniques, for example, can mimic the structure of their template and possess well-interconnected pores, but they often have inadequate mechanical strength [5,6,7]. Meanwhile, in the salt leaching/solvent casting technique, the shape and size of the pore are directly controlled by the porogen/salt used. Another concern about using this technique is that the solvent can be toxic to cells if not completely removed during the process. Adequate fabrication skills are necessary to maintain consistent scaffold architecture when using these traditional techniques [7,8].

Additive manufacturing (AM), a rapid prototyping (RP) technique, also known as three-dimensional printing (3DP), has become a compelling option for producing complex scaffold designs for bone tissue engineering that would be difficult to achieve through traditional means [3,9]. 3DP allows the customisation of scaffold designs to treat the variable needs of patients. Accurate design of the architecture, pore size, and shape can also be achieved [10]. The 3D scaffolds can be constructed using different types of RP techniques such as fused deposition modelling (FDM) (also known as fused filament fabrication, FFF), stereolithography (SLA), selective laser sintering (SLS), particle binding (PB), and inkjet printing (IP). Thermoplastics such as poly(lactic acid) (PLA) and polycaprolactone (PCL) are among the common polymers that have been used to build 3D-printed products using the FDM technique, particularly for BTE applications [5,11].

PLA is a linear aliphatic polyester that has been approved by the Food and Drug Administration (FDA) as a synthetic biodegradable polymer. Despite its advantages features, such as non-toxicity, low immunogenicity, controllable mechanical properties, and predictable degradation rates, the utilisation of PLA in biomedical applications has been restricted due to its hydrophobicity and inability to facilitate cell recognition, leading to poor cell adhesion and proliferation [9,12]. Post-fabrication modification is usually required to improve the performance of PLA scaffolds [13]. Previously, our group successfully developed novel polyelectrolyte multilayers (PEMs) coating onto biodegradable PLA films. This ECM-like coating was created by assembling silicon carbonated hydroxyapatite (SiCHA) dispersed in hyaluronic acid (polyanion) and collagen type I (polycation). SiCHA nanopowders were used as the inorganic component mimicking the composition of native bone [12]. Hyaluronic acid and collagen type I are important components of ECM [7,14].

Another important factor that influences cell response is the size of pores and channels. Channel size plays an important role in cell migration and diffusion of nutrients/waste products [8,10]. For bone tissue engineering purposes, a pore size that is well accepted is in the range of 200–900 μm. However, Holy et al. (2000) proposed that in order to achieve successful bone reconstruction, the 3D substrate should have a macroporous structure with pore sizes ranging from 1.2–2.0 mm to facilitate cell, tissue, and blood vessel in-growth throughout the scaffolds by having a high surface to volume ratio [15]. However, having a 3D scaffold alone is insufficient to induce a 3D pattern of cell ingrowth and differentiation [16,17]. Adding progenitor cells can promote more rapid growth when delivered to the patient. The growth of these 3D constructs requires specialised growth chambers termed bioreactors. These chambers enhance mass transfer throughout the scaffold and provide optimised conditioning of the constructs with tailored biomechanical conditions related to the implant site [18,19]. Previous studies have demonstrated that preconditioning with mechanical forces can lead to remodelling scaffolds and matrices and, therefore, be adapted to the implant site [20].

Bioreactor technologies are the most commonly used for dynamic cell culture studies [19,21]. Ideally, a bioreactor should enable controlled biochemical and/or biological processes [22]. One example of an early bioreactor design still commercially available today is the rotating wall vessels (RWVs) bioreactor, which NASA originally developed for space research. The aim of these bioreactors was to protect cell cultures from the high shear forces generated during the launch and landing of the space transport; however, the system has also been found to provide a suitable growth environment for TE constructs, which requires improved mass transport without mechanical conditioning. In this system, cell constructs are able to rotate in the vessels with minimal disruptive shear stresses, thus simulating microgravity and essentially free from turbulence. RWVs are used to support high-density and large-scale 3D cell cultures and provide a controlled supply of oxygen and nutrients needed for cell growth.

Several studies have shown the effects of microgravity in the culture of osteoblast-like cells [23,24]. However, contradictory results have been described. Some authors reported that microgravity inhibits the proliferation and osteogenic differentiation of mesenchymal stem cells [25,26]. On the other hand, positive impacts of using RWVs bioreactor were demonstrated by others where the improved mass transfer provided by the bioreactor in combination with the appropriate substrate was thought to be a decisive factor for stimulating osteogenic differentiation [24,27]. However, it is known that cell viability and ingrowth are not solely dependent on cell culture conditions. Other important parameters should be considered, such as the physical cues of the scaffolds, in particular the surface composition, roughness, pore/channel size, and porosity. These properties may determine the nutrition exchange throughout the scaffolds, which could greatly affect cell attachment and activity [4,28].

The development of bone tissue relies not only on osteoprogenitor cells, but also on the inclusion of a functional vascular network. This prerequisite is crucial for the survival and integration of constructs within the host tissue. Inadequate vascularisation has been identified as a leading cause of cell death in constructs due to limited nutrient supply, hypoxia, and the accumulation of waste products and non-functional substances. These factors greatly impede the remodelling process and can ultimately lead to the complete failure of the constructs. Despite advancements in various construct fabrication techniques, achieving effective vascularisation remains a major challenge in reconstructing large bone defects.

One extensively explored approach is the in vitro coculture of human umbilical vein endothelial cells (HUVECs) and human mesenchymal stem cells (hMSCs). This approach allows for the concurrent development of a vascular network and the target tissue. However, there is limited knowledge regarding the communication between HUVEC and hMSCs on 3DP constructs with channel design and an innovative coating that mimics the composition of bone tissue.

This study aimed to investigate the fate of human-bone-marrow-derived mesenchymal stem cells (hMSCs) cultured on different structural and functional designs of three-dimensional printed (3DP) hybrid scaffolds in static and dynamic conditions with the use of the rotating wall vessels, RWV. The 3DP scaffolds were initially fabricated using the fused deposition modelling (FDM) technique, followed by surface modification using polyelectrolyte multilayers (PEMs) assembly. The effect of the chemical cues in the culture medium was also investigated in this study by culturing the cellular scaffolds in two different culture mediums, i.e., the osteogenic media (OM) and proliferation media (PM), for both conditions. The optimum 3DP hybrid scaffold was eventually co-cultured using HUVEC/hMSCs in order to provide insight into the crosstalk between the cells by means of the early osteogenic and angiogenic expression as well as the secretion of the pro-angiogenic growth factors in particular vascular endothelial growth factor (VEGF) and platelet-derived growth factor (PDGF).

## 2. Materials and Methods

### 2.1. Materials

All reactants, i.e., calcium nitrate tetrahydrate, Ca(NO_3_)_2_^.^ 4H_2_O (99.0% pure), di-ammonium hydrogen phosphate, (NH_4_)_2_HPO_4_ (98.0% pure), ammonium hydrogen carbonate, NH_4_HCO_3_ (99.0% pure), and silicon tetra acetate, Si(CH_3_COO)_4_ (98.0% pure), were purchased from Sigma-Aldrich (UK). Poly (lactic acid) resin (Product code: 4032 D) was purchased from NatureWorks^®^ LLC (USA). The culture media was prepared using 4.5 g/L Dulbecco’s Modified Eagle Medium, DMEM (Lonza, UK), L-glutamine (Lonza, UK), Penicillin-Streptomycin (Lonza, UK), Foetal Bovine Serum (Biosera labtech, UK), Dexamethasone (Sigma-Aldrich, UK), Ascorbic Acid (Analar, UK), and β-Glycerophosphate (Sigma-Aldrich, UK). Human mesenchymal stem cells (hMSCs) isolated from a bone marrow aspirate obtained from a 24-year-old male (Lonza, UK) at passage zero (P0) were expanded until passage two (P2) when the required cell number was obtained. The cell number was calculated using a cell counter.

### 2.2. Preparation of SiCHA Nanopowders

SiCHA nanopowders were chemically synthesised using the nanoemulsion method at ambient temperature, as described in our previous report published elsewhere. The as-synthesised powders were then calcined at 500 °C in an air atmosphere with a heating rate of 10 °C/min and one hour of soaking using a chamber furnace (Carbolite CWF-B 12/13, UK). SiCHA nanopowder was selected due to its composition closely matching the physiological range of ionic substitutions found in bone minerals, containing 3.98 wt% carbonate and 0.45 wt% Si-substituted into the HA host structure. This makes it the most favourable environment for the growth of hMSCs in vitro, supporting culture for up to 21 days, compared to the prepared CHA nanopowders, as reported in our previous study [29].

### 2.3. 3D Printing

Three different structural designs of the scaffolds were investigated, namely four channels (4C), two channels (2C), and mesh scaffolds. The 3D scaffolds were fabricated using Poly (lactic acid) filament via the fused deposition modelling (FDM) method. Scaffolds were printed using Ultimaker 2 from Ultimaker (UK). Before printing, the scaffolds were first designed using Autodesk Inventor Professional 2014—the computer-aided design (CAD) drawings for 4C and 2C scaffolds, with the diameter of each channel being about 1.5 mm. The scaffolds were then printed at the optimised speed of 40% at 210 °C for the first four layers from the bottom, a slightly slower speed of 25% for the middle layers (5–14 layers from the bottom), at 200 °C, and finally, the last five layers were printed at a speed of 40% at 210 °C. The infill density was kept constant for each layer at 75%.

### 2.4. Fabrication of 3DP Hybrid Scaffolds

The fabricated scaffolds were then surface-modified by a chemical route. The surface of the 3DP PLA scaffolds was modified by introducing amino functional groups through aminolysis. To create the 3DP hybrid scaffolds, the printed scaffolds were deposited with innovative coating materials. The surface-modified 3DP PLA scaffolds were coated with five bilayers (5-BL) of the newly developed coating materials assembled by SiCHA nanopowders in hyaluronic acid (polyanion) and collagen type I (polycation). A mixture of EDC/NHS was used to crosslink the polyelectrolyte layers. In brief, the aminolysed 3DP PLA scaffolds with positively charged surfaces were first immersed in the polyanion solution for 15 min, then rinsed in ultrapure water (pH 5.0). After rinsing, the films were immersed in the polycation solution for another 15 min. They were then washed again in fresh ultrapure water (pH 5.0) to remove any unbound materials and prevent contamination of the polyelectrolyte solution. Finally, an EDC/NHS solution was introduced as the final step of each multilayer coating, followed by a rinse in ultrapure water. The coating steps were repeated five times. Details on the coating procedure were described in our previous work [12].

### 2.5. Cell Seeding on 3DP Hybrid Scaffold

All scaffolds were sterilised three times in the UV Chamber for 90 s each cycle, followed by pre-wetting in PM for three hours before cell seeding. Commercial hydroxyapatite (HA) scaffolds were used as control samples. These scaffolds required longer soaking in PM (72 h), as recommended by the manufacturer (Ceramisys, UK). A concentrated cell suspension ≤ 20 μL containing 1 × 10^5^ hMSCs was seeded on the sterilised 3DP hybrid scaffolds. A rotating wall vessel, RWV (Synthecon Inc., Luxembourg), was used to culture the cellular scaffolds in dynamic condition. The speed of the rotating bioreactor was set at 20 rpm. Two test groups were then established. Half of the scaffolds were transferred to fresh 24-well plates and incubated statically after adding 1.5 mL media to each well; the other cellular scaffolds were directly transferred to the RWV chambers containing 60 mL complete medium (either osteogenic or proliferation media). The static and dynamic test groups were then divided into groups of different culture mediums used, namely (1) osteogenic media (OM) and (2) proliferation media (PM).

### 2.6. In Vitro Biocompatibility Study on 3DP Hybrid Scaffold

In vitro assessments, including cell viability, proliferation, metabolic activity, and early osteogenic differentiation, were conducted to identify the best structural design and functional 3DP hybrid scaffolds. Commercial hydroxyapatite (HA) scaffolds were used as the experimental control since this product is commercially available and has supporting clinical data. It should be noted that the clinical data are not shown in this study since they are highly confidential. For the biochemical assays, the cellular scaffolds were rinsed with PBS, trypsinised, washed again with PBS, and finally frozen at −80 °C in 1 mL of dH_2_O at the time point. After three cycles of freeze/thaw, the samples were ready for the cytotoxicity assessment. All biochemical assays were read using a Synergy II BioTek plate reader.

### 2.7. Cell Viability

The cell viability was observed using Confocal Laser Scanning Microscope (CLSM) Olympus Fluoview FV 1200 with Fluoview Version 4.1 software (Olympus, UK) using the Live/Dead Assay Kit (Invitrogen, UK) according to the manufacturer’s instructions. Calcein-AM ester was used to label viable cells (green) fluorescently; the nucleus of dead cells was labelled with Propidium Iodide (red). Briefly, cell culture media was removed from samples. The cellular scaffolds were washed with PBS, immersed in the staining solution containing 10 µM Calcein-AM and 1 µM Propidium Iodide in PBS, and incubated at 37 °C for 20 min in the dark. The samples were washed once with 1.0 mL of PBS and immediately imaged using CLSM.

### 2.8. Cell Proliferation

The Quant-iTTM Picogreen^®^ dsDNA assay kit (Invitrogen, UK) was used according to the manufacturer’s instructions. The Picogreen solution was prepared as 1:200 dilutions in 1 × Tris-EDTA (TE) buffer. Ranges of DNA dilutions (0–2 μg/mL) were used to construct a standard curve. Then, 100 µL of cell lysate or DNA standard was placed in each well of a 96-well plate, followed by 100 µL of Picogreen reagent. This was incubated at room temperature in the dark for 5 min before reading the fluorescence at 485/535 nm (excitation/emission).

### 2.9. Lactate Dehydrogenase Assay (LDH)

The LDH assay is a reliable colourimetric assay that quantifies the LDH release into the media from damaged cells as a biomarker for cellular cytotoxicity. An LDH assay kit (Thermo Fisher, UK) was used according to the manufacturer’s instructions. Briefly, 50 μL of media from either the well or RWVs were transferred into a 96-well plate, then incubated for 45 min at 37 °C with 5% CO_2_. About 50 μL of the reaction mixture was added to the relevant well. Samples were incubated for 30 min in the dark at room temperature. Finally, 50 μL of stop solution was added to each well, followed by mixing with gentle tapping. Absorbance readings were taken at 530/590 nm (excitation/emission). LDH activity and percentage of cytotoxicity (%cytotoxicity) of each sample were calculated using the following equations:

LDH Activity = Abs. value (490 nm) − Abs. value (680 nm)(1)

(2) $$\%\;\mathrm{Cytotoxicity}=\frac{\text{Compound-treated LDH activity}-\text{Spontaneous LDH activity}}{\text{Max LDH activity}-\text{Spontaneous LDH activity}}\times 100\%$$

### 2.10. Total Protein

The levels of total protein were quantified using Bradford reagent (Sigma-Aldrich, UK). Ranges of protein standard solutions (0–2 mg/mL) were prepared by dissolving Bovine Serum Albumin, BSA (Sigma-Aldrich, UK) in distilled water. For total protein assay, 50 μL samples or standards were placed in each well of 96-well plates, followed by the addition of 50 µL of Bradford reagent. Samples were incubated for 5 min at room temperature before reading the absorbance level at 595 nm.

### 2.11. Cell Activity

Alkaline phosphatase (ALP) activity was obtained from a 4-Methylumbelliferyl phosphate (4-MUP, Sigma-Aldrich, UK) reaction. Ranges of 4-Methylumbelliferone (4-MU, Sigma-Aldrich, Switzerland) dilutions (0–2 μg/mL) were used to construct a standard curve. In total, 50 µL of the cell lysate from each sample or standard of 4-MU and 50 µL of 4-MUP was added into the relevant well of a 96-well plate, followed by incubation at 37 °C for 90 min. To terminate the reaction, 100 µL of 1 × TE was added, and the fluorescence reading was taken at 360/440 nm (excitation/emission).

### 2.12. Alkaline Phosphatase (ALP) Staining

The pre-cursor of early bone mineralisation was stained using an ALP detection kit purchased from Merck Millipore (UK). Scaffolds were transferred to fresh 24-well plates and rinsed once with PBS. Scaffolds were fixed in 4% Paraformaldehyde (Sigma-Aldrich, UK for 90 s, followed by washing in TBST solution (20 mM Tris-HCl, pH 7.40, 0.15 M NaCl, 0.05% Tween-20). The working solution was prepared according to the manufacturer’s instruction with 2:1:1 ratios of Fast Red Violet solution: Naphthol AS-BI phosphate solution: dH_2_O. In total, 500 μL of the working solution was added to each well and left in dark condition at room temperature for 30 min. The scaffolds were carefully rinsed twice with distilled water. The stained scaffolds were imaged under a dissection microscope (Leica, UK).

### 2.13. Micro-Computed Tomography (μ-CT) Analysis

Scaffolds were fixed in 1.0 mL of 10% formalin (Sigma-Aldrich, UK) at 4 °C overnight. X-ray micro-computed tomography, μ-CT (microCT40, Scanco Medical, Switzerland) with a beam energy of 55 kVp, beam intensity of 145 μA, 200 ms integration time, and spatial resolution of 10 μm was used to observe any sign of early formation of bone mineralisation on the cellular scaffolds. The total volume (TV) value was obtained at a threshold of 55, while the bone volume (BV) value was generated at a higher threshold of 120. The estimated percentage of bone mineralisation (% BV/TV) was obtained by normalising the values of BV at a threshold of 120 over TV at a threshold of 55.

### 2.14. Co-Culture

Human umbilical vein endothelial cells, HUVECs (Life Technologies, UK) at passage three (P3) were cultured in complete Endothelial Media (EM) consisting of Medium-200 with Low Serum Growth Supplement (LSGS) containing 2% *v*/*v* FBS, 1 µg/mL hydrocortisone, 10 ng/mL human epidermal growth factor, 3 ng/mL basic fibroblast growth factor, and 10 µg/mL heparin. Both Medium-200 and LSGS kits were purchased from Thermo-Fisher Scientific (UK). hMSCs (Lonza, USA) at the same passage number (P3) was expanded in proliferation media. Both cell types were cultured in standard cell culture flasks incubated at 37 °C with 5% CO_2_ and 95% relative humidity for about 10 days till 80–90% confluent levels were achieved. Both HUVECs and hMSCs were expanded up to passage three (P3) and used for the study at passage four (P4).

### 2.15. Labelling with Fluorescent Dyes

Red fluorescent dye, PKH26 (Paul Karl Horan 26, Sigma-Aldrich, UK) was used to label the HUVECs, while hMSCs were labelled using the Cell Tracker Blue CMAC (7-amino-4-chloromethyl coumarin, Molecular Probes, Life Technologies, USA). Labelling was carried out according to the manufacturer’s instructions with 4 µL/mL PKH26 (red dye) in Dilute C. Briefly, after cell counting, 2.0 × 10^6^ cells were resuspended in complete media. The cell pellet obtained was then washed with serum-free media and resuspended in complete media; these steps were repeated three times. After pelleting, 1.0 mL of Dilute C was added directly to the cell suspension and mixed well; subsequently, 4 μL of red dye was then added to the cell solutions, followed by incubation at 37 °C for 10 min. To ensure the cells were properly labelled, the unbound dye was blocked using 1% BSA, followed by incubation at room temperature for one minute. The cell solution was resuspended and subsequently washed three times with complete EM. To ease HUVECs proliferation, Matrigel from BD Bioscience (USA) was then added to the cell solution. This was then divided into two groups, each aliquot containing 1.2 × 10^6^ and 0.6 × 10^6^ cells for the HUVECs control and co-culture samples. A similar procedure was used to label the hMSCs with CMAC (blue dye). The concentration of the blue dye used was 4 µL/mL blue dye in serum-free media. For hMSCs, the cell solution was incubated at 37 °C for 30 min after adding the blue dye. The cell solution was washed with PM three times. Finally, 2.0 mL of fresh OM was added to the cell solution. The total amount was divided into two groups, with each aliquot containing 1.2 × 10^6^ and 0.6 × 10^6^ cells for the hMSCs control and co-culture samples, respectively.

### 2.16. Seeding Optimum Hybrid Scaffolds

For co-culture samples, a 1:1 of HUVECs:hMSCs cell ratio was used. The channels of the hybrid scaffolds were directly seeded with 6.25 × 10^3^ of HUVECs in Matrigel per channel. Scaffolds were then incubated for 30 min at 37 °C to enhance the gelation of Matrigel. The scaffolds were then turned onto the opposing side, and the process repeated. The cellular scaffolds were cultured for 3 days in complete EM at 37 °C with 5% CO_2_ and 95% relative humidity before adding hMSCs. Each side of the scaffold was seeded with 2.5 × 10^4^ labelled hMSCs. Each side was subjected to 3 h of incubation to allow the hMSCs to adhere to the surface, followed by repeating the seeding procedure on the other side. Control scaffolds were seeded with a single cell type, either HUVECs or hMSCs alone. The same seeding protocols were used. For ECs controls, 5 × 10^4^ of labelled HUVECs were seeded in the channels and cultured in EM. Meanwhile, 5 × 10^4^ labelled hMSCs were seeded on the surface of the scaffolds and cultured in OM, acting as the hMSCs control samples. The medium was refreshed every 3 days.

### 2.17. Enzyme-Linked Immunosorbent Assay for PDGF-BB and VEGF

The levels of platelet-derived growth factor-BB (PDGF-BB) and vascular endothelial growth factor (VEGF) were quantified using enzyme-linked immunosorbent assay (ELISA) kits purchased from R&D Systems (UK). The cell culture media of HUVECs alone, hMSCs alone, and a co-culture of HUVECs/hMSCs were collected on days 3 and 10 of culture. The culture medium (without cells) of EM, OM and mix media of EM:OM was used as the experimental control for both immunoassays. Assays were performed according to the technical datasheet provided by the manufacturer, and the absorbance was read at 450 nm.

### 2.18. Statistical Analysis

Quantitative data were presented as means ± standard deviation (SD). A Kolmogorov–Smirnov test, with Dallal–Wilkinson–Lillie for a corrected *p*-value, was performed to determine the normal distribution of the data (recommended for small n data analysis). A two-way ANOVA with multiple comparisons Tukey test was performed to define the best scaffold after 21 days under different culture conditions and culture medium. A two-way ANOVA with multiple comparisons Tukey test was performed to compare the level of PDGF and VEGF expressions secreted by the co-culture and their monoculture systems at each time point. Statistical significance was considered for *p* ≤ 0.05 (*), *p* ≤ 0.01 (**), *p* ≤ 0.001 (***) and *p* ≤ 0.0001 (****). All statistical analyses were performed using GraphPad Prism 7 software. For bioassays, tests were performed on *n* = 3 in duplicates. For μ-CT analysis, *n* = 3 and *n* = 2 were used for imaging.

## 3. Results

### 3.1. Cell Viability

**Figure 1:**
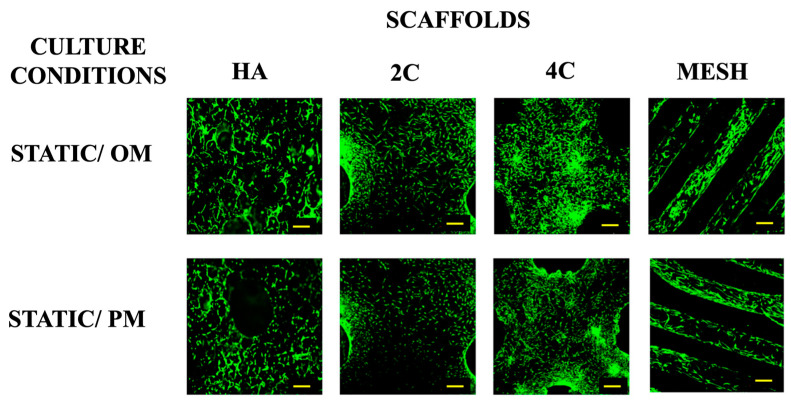
Viability of hMSCs seeded on all four scaffold designs cultured under static condition OM and PM after 1 day (for 3DP hybrid scaffolds) and 3 days (for HA scaffolds). Yellow scale bar = 500 μm.

Live/dead staining was performed before the cellular scaffolds were transferred into the RWV system to confirm that the cells were attached to scaffolds before any stimulation was applied (Figure 1). This confirmed that scaffolds were comparably seeded before applying the dynamic culture, ensuring that any differences could be attributed to the culture condition rather than differences in the initial cell seeding density. Higher proportions of viable cells were found on 3DP hybrid scaffolds compared to the HA scaffolds one day after seeding. This indicates that the layer-by-layer coating of SiCHA nanopowders embedded in the hyaluronic acid and collagen type I on the 3DP PLA scaffolds provides a favourable environment for supporting cell attachment than HA alone.

**Figure 2:**
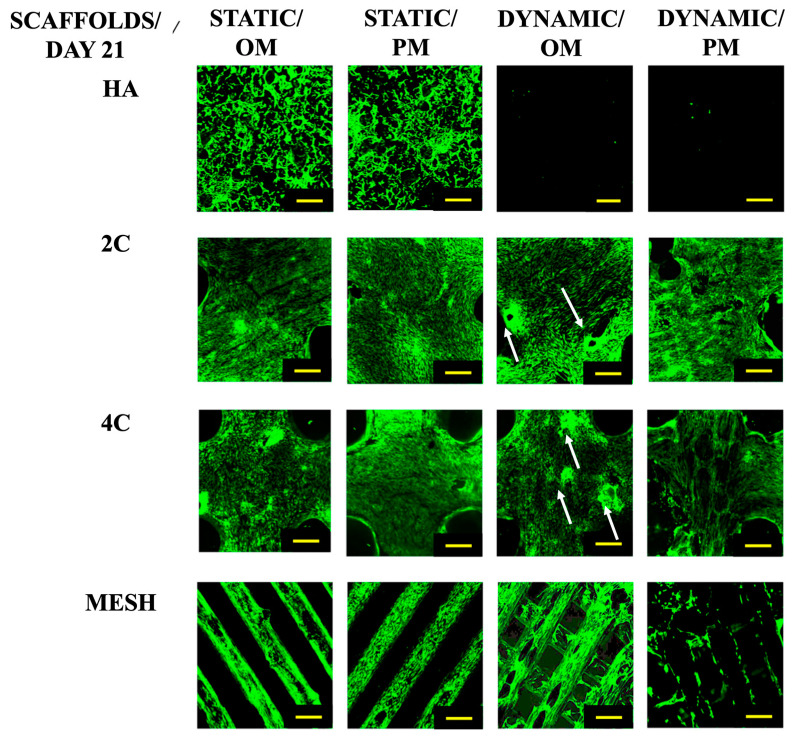
Cell viability for different scaffold designs after 21 days cultured under static and dynamic conditions in OM and PM. Yellow scale bar = 500 μm.

After a 21-day culture period (Figure 2), it was evident that the cells could only proliferate within the HA scaffolds under static conditions. While the cells seeded on the 3DP hybrid scaffolds remained viable across all culture conditions. When grown statically in various culture media, the cells on the 3DP hybrid cellular scaffolds successfully attached and proliferated along the struts of the meshes. Additionally, these scaffolds demonstrated the formation of bone-like nodules in the Dynamic/OM condition. However, in Dynamic/PM condition, cell detachment occurred from the surface of the scaffold due to over-confluency.

In contrast, the cells seeded on HA scaffolds did not survive when exposed to the RWV bioreactor, irrespective of the culture medium used. These findings clearly demonstrate the effectiveness of the coating developed in this study, which positively impacts cell activity and growth.

### 3.2. Cell Proliferation

**Figure 3:**
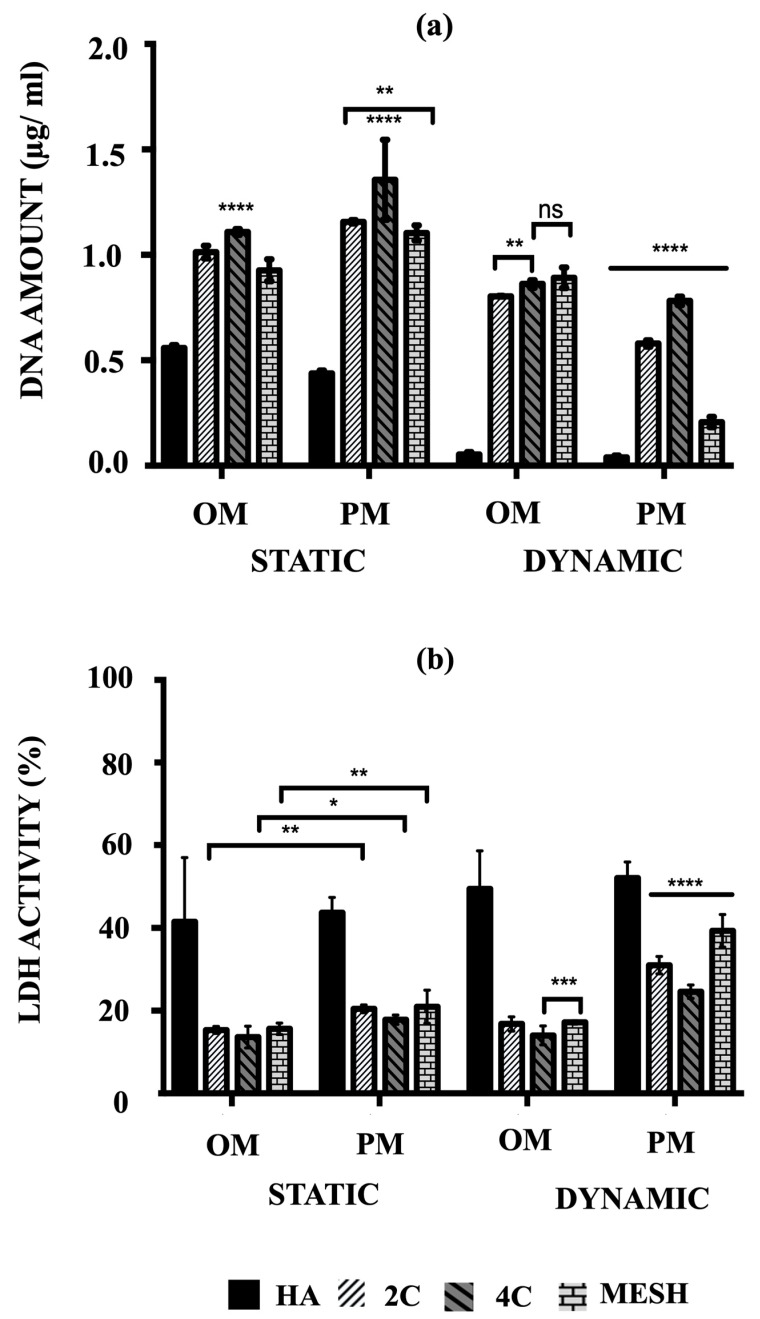
(**a**) Comparison of the amount of DNA associated with hMSCs and (**b**) the percentages of LDH activity on HA, 2C, 4C and mesh scaffolds in different culture conditions after 21 days (ns ≥ 0.05; * *p* ≤ 0.05, ** *p* ≤ 0.01, *** *p* ≤ 0.001, **** *p* ≤ 0.0001).

The cell proliferation of hMSCs on HA, 2C, 4C, and mesh scaffolds cultured in different conditions was assessed by their amounts of DNA after 21 days (Figure 3a). The main objective was to quantitatively determine which scaffold design and culture condition/medium composition could best facilitate rapid cell proliferation, considering the remarkable cell viability enhancement offered by the 3DP hybrid scaffolds.

After 21 days, mean DNA concentrations were significantly higher for static conditions, both OM and PM, for the tested scaffold designs (*p* ≤ 0.05) and differed between scaffold designs (*p* ≤ 0.0001). There was a significant interaction between culture condition/media and scaffold designs (*p* ≤ 0.05), suggesting that for all tested scaffold designs, significantly higher mean DNA concentrations were obtained in the static condition (*p* ≤ 0.0001). Culturing the 3DP hybrid scaffolds in dynamic conditions for both OM and PM resulted in significantly lower DNA concentrations than static conditions, particularly in Dynamic/PM (*p* ≤ 0.0001 for each). Compared to the control (HA cellular scaffolds), all 3DP hybrid scaffolds showed significantly higher DNA concentrations regardless of the culture condition/media (*p* ≤ 0.0001 for each). When comparing the 3DP hybrid scaffold designs, the highest mean DNA concentrations were observed on 4C scaffolds in static for both OM and PM compared to 2C and mesh scaffolds (*p* ≤ 0.0001 for each). When cells were cultured in Static/PM, significantly higher mean DNA concentration was obtained on 2C scaffolds (1.16 ± 0.03 μg/mL) than mesh (1.10 ± 0.01 μg/mL) (*p* = 0.0064). No significant differences were observed when cells were cultured on 4C (0.87 ± 0.01 μg/mL) and mesh (0.89 ± 0.02 μg/mL) scaffolds in Dynamic/OM (*p* = 0.6283). In Dynamic/PM, culturing cells on 4C scaffolds (0.78 ± 0.01 μg/mL) resulted in the highest mean DNA concentration, while the lowest mean DNA concentration was obtained by culturing on mesh scaffolds (0.21 ± 0.01 μg/mL) (*p* ≤ 0.0001 for each). Overall, 4C scaffolds in Static/PM revealed the highest mean DNA concentrations compared to all other investigated groups (scaffolds designs and culture condition/media) on day 21 (*p* ≤ 0.0001 for each).

### 3.3. Cytotoxicity Percentages

The cytotoxicity percentages of hMSCs cultured on both HA and 3DP hybrid scaffolds under various culture conditions were assessed by measuring the lactate levels excreted into the culture medium, as per Equation (1). Subsequently, these cytotoxicity percentages (%LDH activity) were calculated using Equation (2). Higher percentages (e.g., 100%) indicate that the scaffolds or conditions were toxic to the cells and led to or had the potential to cause cell death. Conversely, lower percentages (e.g., 0%) indicate that the conditions were favourable for maintaining cell viability.

The highest mean %LDH activity was obtained in Dynamic/PM for all tested scaffold designs (*p* ≤ 0.0001) and differed between scaffold designs (*p* ≤ 0.0001) at day 21 (Figure 3b). There was a notable interaction between culture condition/media and scaffold designs (*p* ≤ 0.05), indicating that for all tested scaffold designs, significantly higher mean %LDH activity was obtained in PM (*p* ≤ 0.0001). In contrast, all 3DP hybrid scaffolds consistently exhibited significantly lower %LDH activity compared to HA cellular scaffolds (control), regardless of the culture condition/media (*p* ≤ 0.0001 for each). This observation is opposite to the trend seen in the quantified DNA amounts.

When comparing the 3DP hybrid scaffold designs, no significant differences in mean %LDH activity were found when 2C, 4C, and mesh cellular scaffolds were cultured in static conditions, in both culture media (*p* ≤ 0.0001 for each). However, under Dynamic/OM conditions, 4C scaffolds (14.03 ± 0.92%) showed significantly lower mean %LDH activity than 2C (16.81 ± 0.71%) (*p* ≤ 0.0001) and mesh scaffolds (17.18 ± 0.17%) (*p* = 0.0008). A similar trend was observed in Dynamic/PM, where significantly lower %LDH activity was obtained on 4C scaffolds (24.60 ± 0.67%) compared to 2C (31.01 ± 0.86%) and mesh (39.30 ± 1.62%) scaffolds (*p* ≤ 0.0001 for each).

### 3.4. Total Protein Production

**Figure 4:**
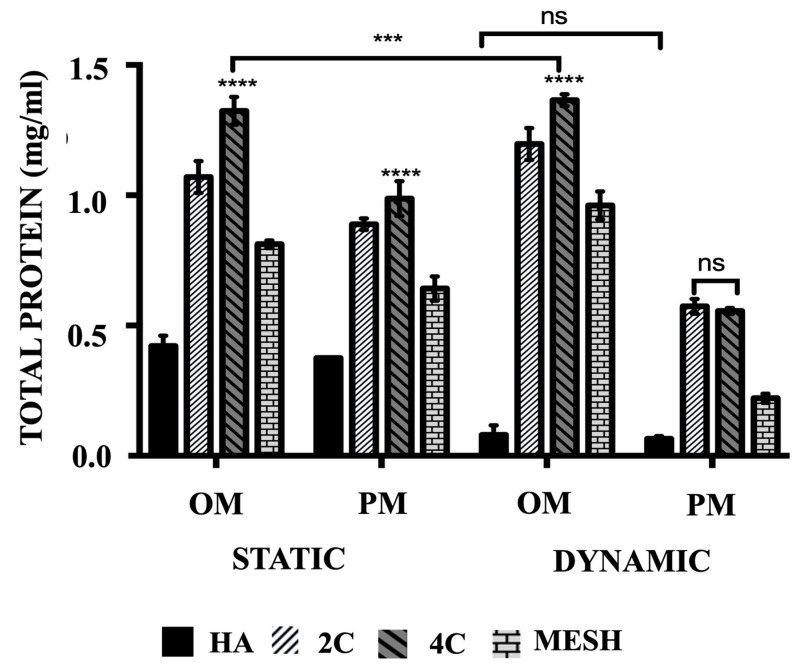
The comparison of the total protein produced of hMSCs after 21 days cultured on HA, 2C, 4C, and mesh scaffolds in different culture conditions. (ns ≥ 0.05; *** *p* ≤ 0.001, **** *p* ≤ 0.0001).

The mean total protein concentrations (Figure 4) are significantly higher when cultured in OM than PM both in static and dynamic conditions for all tested scaffold designs (*p* ≤ 0.05) and differed between scaffold designs (*p* ≤ 0.0001). Culturing HA scaffolds in dynamic conditions using OM or PM did not lead to any significant differences (*p* = 0.9643). In comparison to the control group (HA cellular scaffolds), all 3DP hybrid scaffolds exhibited significantly higher mean total protein concentrations, regardless of the culture condition or media used (*p* ≤ 0.0001 for each). Further analysis of the various 3DP hybrid scaffold designs revealed that cells grown on 4C scaffolds demonstrated the highest mean total protein levels in static cultures, both in OM (1.32 ± 0.02 mg/mL) and PM (0.99 ± 0.02 mg/mL) media, as well as in Dynamic/OM (1.37 ± 0.01 mg/mL) when compared to 2C scaffolds cultured in Static/OM (1.07 ± 0.03 mg/mL), Static/PM (0.89 ± 0.01 mg/mL) and Dynamic/OM (1.20 ± 0.02 mg/mL) (*p* ≤ 0.0001 for each). However, no significant differences were observed between 2C (0.57 ± 0.01 mg/mL) and 4C (0.56 ± 0.01 mg/mL) scaffolds when cultured in Dynamic/PM (*p* = 0.8652). Among the 3DP hybrid scaffolds, cells cultured on mesh scaffolds exhibited the lowest mean total protein concentrations in all culture conditions and media (*p* ≤ 0.0001 for each).

### 3.5. Cell Activity

**Figure 5:**
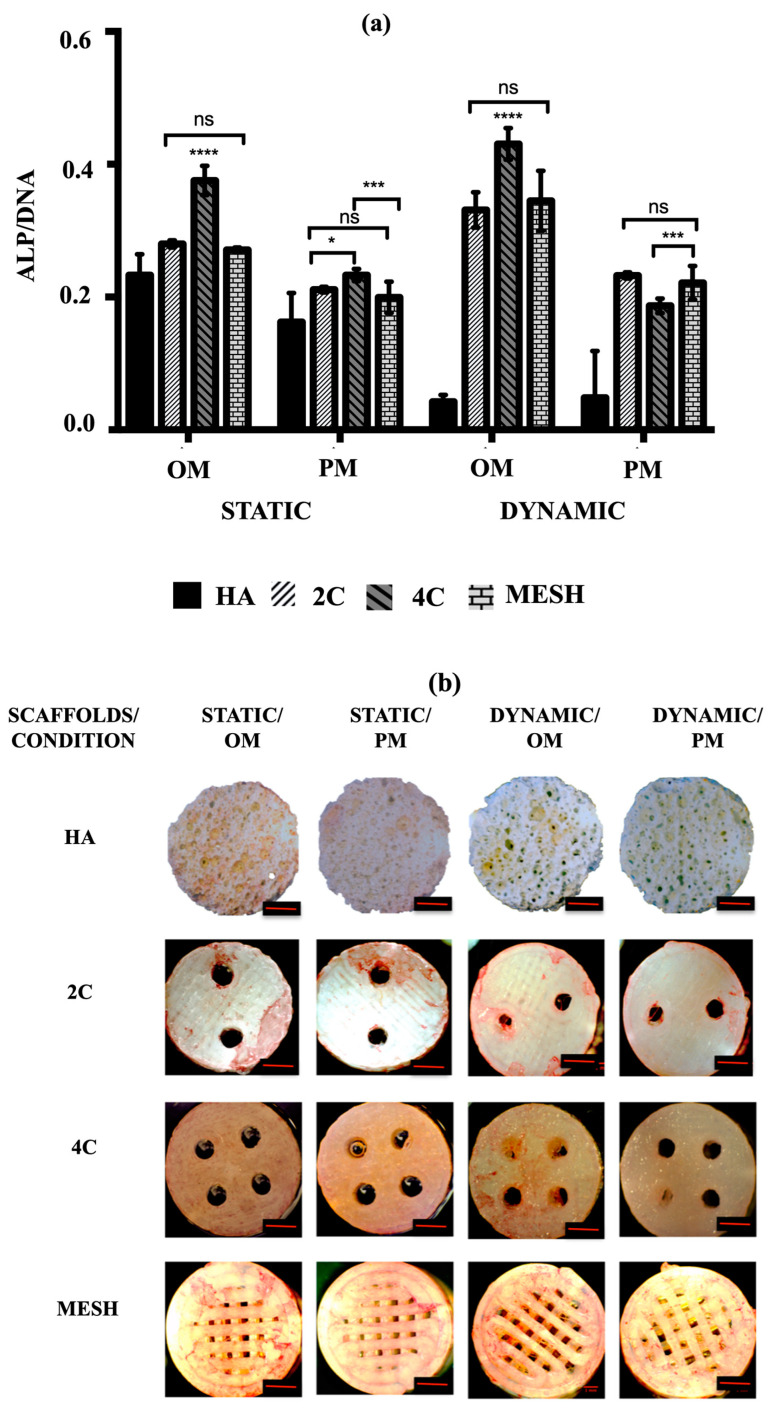
(**a**) ALP activity of hMSCs after 21 days cultured on HA, 2C, 4C, and mesh scaffolds in different culture conditions (ns ≥ 0.05; * *p* ≤ 0.05, *** *p* ≤ 0.001, **** *p* ≤ 0.0001). (**b**) ALP staining for different scaffold designs after 21 days cultured under static and dynamic conditions in OM and PM. Red scale bar = 1 mm.

To assess the early osteogenic differentiation potential of hMSCs on various scaffold designs under different culture conditions, the mean ALP activity was measured after 21 days. The goal was to identify the scaffold design and culture condition/medium that could promote the fastest osteogenic differentiation. According to the data presented in Figure 5a, the mean ALP activity was significantly higher in cultures using OM compared to PM in both static and dynamic conditions for all tested scaffold designs (*p* ≤ 0.0001). In addition to demonstrating enhanced cell activities and growth, all 3DP hybrid scaffolds displayed markedly higher mean ALP activity than HA in all instances (*p* ≤ 0.0001 for each). Nevertheless, when HA scaffolds were cultured in dynamic conditions, no significant differences in mean ALP activity were observed between OM (0.04 ± 0.01) and PM (0.04 ± 0.02) (*p* = 0.9699). Among the 3DP hybrid scaffold designs, cells grown on 4C scaffolds demonstrated the highest mean ALP activity in OM for both static (0.38 ± 0.01) and dynamic (0.43 ± 0.01) conditions (*p* ≤ 0.0001 for each). However, when 4C scaffolds were cultured in Dynamic/PM (0.19 ± 0.01), significantly lower mean ALP activity was observed compared to 2C (0.23 ± 0.01) (*p* ≤ 0.0001) and mesh scaffolds (0.22 ± 0.01) (*p* = 0.0002). On the other hand, there were no significant differences between 2C and mesh scaffolds when cultured in Dynamic/PM (*p* = 0.7464).

Alkaline phosphatase (ALP) staining was then used to qualitatively identify the expression of osteoblastic phenotype. A positive ALP stain appeared red. No positive ALP stains were observed on the HA scaffolds for all culture conditions after 21 days (Figure 5b). While positive ALP stains were obtained for the 3DP hybrid scaffolds, particularly with the presence of OM. In Static/OM, the 4C cellular scaffolds showed the most homogenous ALP expression across the entire scaffolds compared to the 2C and the mesh scaffolds. When the cellular scaffolds were exposed to the dynamic condition in the RWV, the ALP expression of the 4C and mesh scaffolds seemed higher than the 2C scaffold.

### 3.6. Formation of the Mineralised Matrix

**Figure 6:**
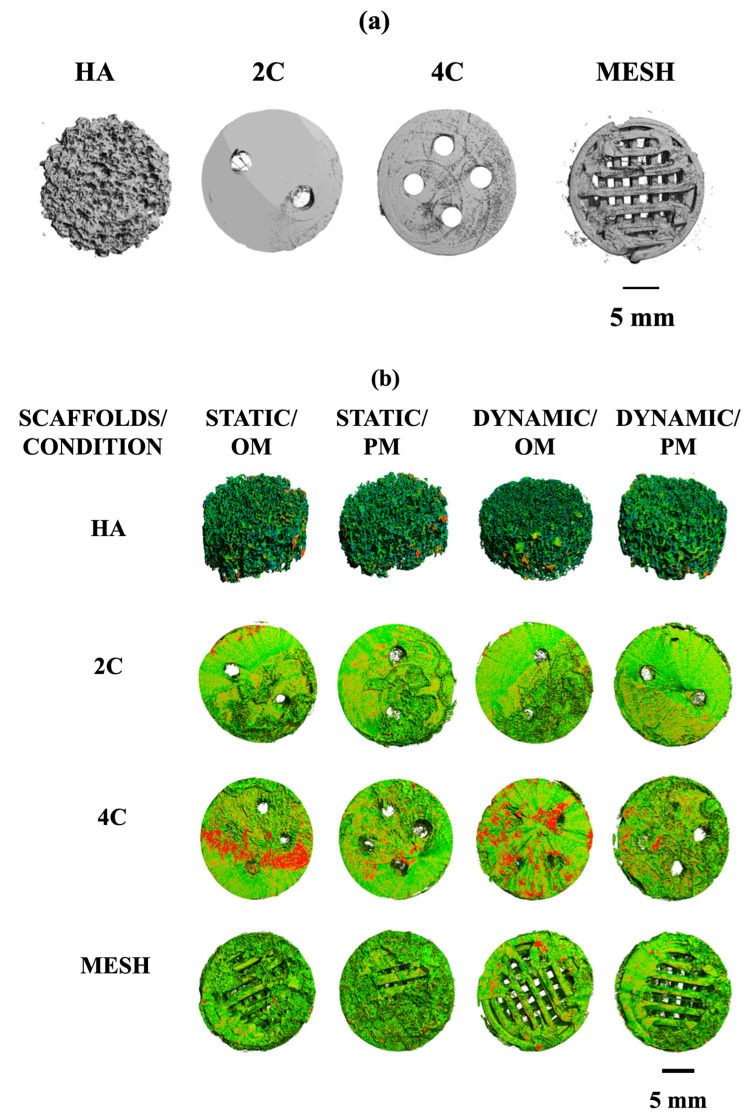
(**a**): X-ray Micro-CT of different structural designs of dry scaffolds (before seeding). (**b**): Density maps of HA, 2C, 4C, and mesh scaffolds after 21 days in different culture conditions. This figure demonstrated the comparisons of the formation of the mineralised matrix (designated by red area) by hMSCs on different scaffold designs under different culture conditions.

Micro-computed tomography (μ-CT) analysis evaluated the mineralised matrix formation on different scaffold designs. Prior to cell seeding, all scaffolds were scanned at different thresholds. HA scaffold is denser in nature and was scanned at a higher density threshold compared to the hybrid scaffolds, which were fabricated by the fused deposition modelling technique using poly (lactic acid) scaffolds coated with 5-BL of SiCHA in hyaluronan/SiCHA in collagen type I and EDC/NHS coupling agent Figure 6a.

By comparing the four scaffold designs, it is clearly seen that 4C cellular scaffolds in osteogenic media for both culture conditions exhibited a higher portion of the denser area, which was assumed to be the mineralised matrix. In fact, a more mineralised matrix was detected as the 4C cellular scaffolds were cultured in the dynamic environment (Figure 6b).

Quantitative μ-CT analysis was then performed to determine the changes in the total volumes and percentages of mineralisation formed on the cellular scaffolds. Table 1 represents the total volume of the dry scaffolds (before seeding) and the cellular scaffolds under different culture conditions after 21 days. Overall, the total volumes of all the cellular scaffolds increased in all culture conditions over 21 days. These results are consistent with the density maps shown in Figure 6b. The 4C cellular scaffolds in all culture conditions exhibited the highest increase in the total volumes as compared to other scaffold designs, except mesh scaffolds in Dynamic/OM. All cellular scaffolds cultured in osteogenic media revealed higher total volume than in proliferation media for both static and dynamic conditions. This became more apparent when the cellular scaffolds were cultured in a dynamic environment. For instance, mesh cellular scaffolds were found to have the highest total volume when cultured in Dynamic/OM. However, HA cellular scaffolds exhibited the lowest percentage of mineralisation as compared to the 3DP hybrid scaffolds under all culture conditions after 21 days.

Regardless of the scaffold designs, the 3DP hybrid scaffolds demonstrated superior performance over HA scaffolds in producing mineralised matrix, especially under osteogenic conditions (Table 2). Our finding highlighted that the novel composition of the 3DP hybrid scaffolds built up layer-by-layer of SiCHA nanopowders in hyaluronic acid and collagen type I significantly impacts cell behaviour compared to the control HA scaffolds. The improvements in cell attachment and growth characteristics signify that the composition and fabrication route confer 3DP hybrid scaffolds with enhanced surface and biochemical cues that promote a highly favourable cell–material interaction.

**Figure 7:**
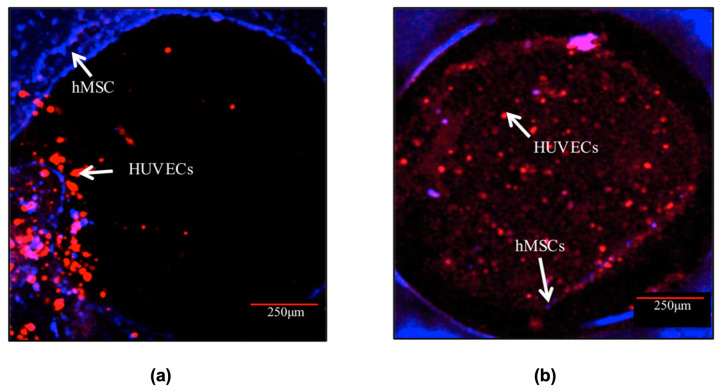
Cell morphology of the co-culture system in the channel of the 4C scaffolds after (**a**) 3 and (**b**) 10 days post-hMSCs addition. HUVECs (labelled in red) were distributed in the entire channels after 10 days post hMSCs (labelled in blue) addition. Scale bar = 250 μm.

In progressing further, the 4C hybrid scaffold was chosen as the most effective design among the tested scaffolds in investigating the potential for in vitro pre-vascularisation of bone TE constructs. The CLSM images (Figure 7) exhibited that both cell types remained viable and increased in cell number over the entire scaffolds throughout the culture. It was observed that after 3 days post-hMSCs addition, HUVECs formed aggregate-like structures in the channels. The hMSCs, on the other hand, remained at the periphery of the channels. After 10 days in culture, HUVECs spread out and organised themselves throughout the channels. HUVECs in the channels also migrated to the surface and towards the hMSCs especially the cells close to the periphery of the channels. In addition, a small number of hMSCs migrated Into the channels.

**Figure 8:**
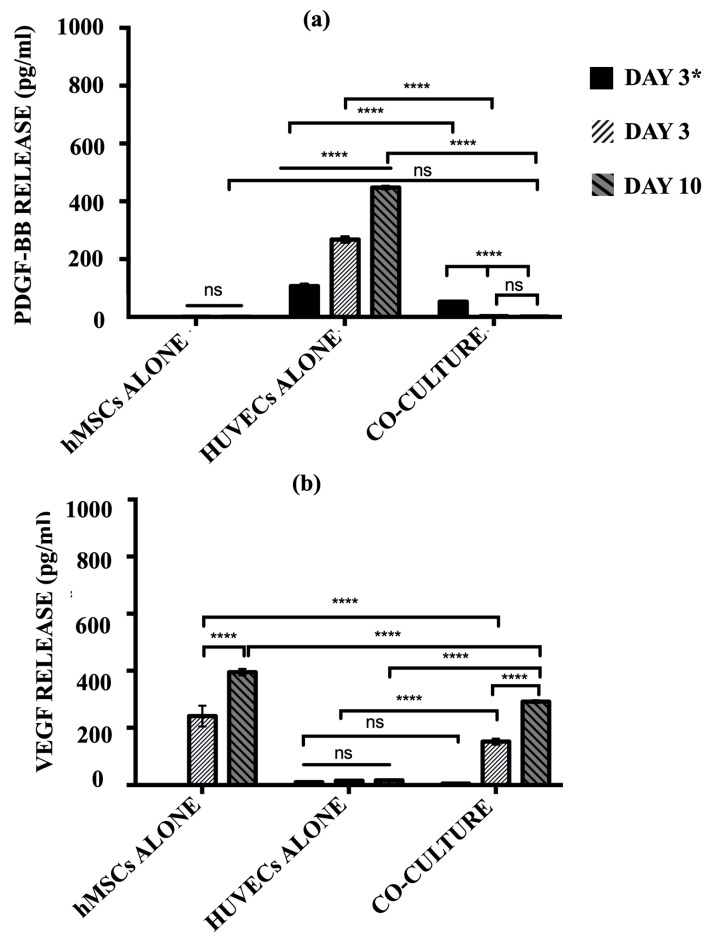
The level of (**a**) PDGF-BB and (**b**) VEGF release by hMSCs alone, HUVECs alone, and co-culture model after day 3 HUVECs seeding only and day 6 and 10 post-hMSCs additions. (ns ≥ 0.05; **** *p* ≤ 0.0001).

The secretion of pro-angiogenic signalling molecules, PDGF-BB and VEGF, into the cell culture media by hMSCs alone, HUVECs alone, and the co-culture model was quantified using ELISA (Figure 8). HUVECs alone group produced a significantly high level of PDGF-BB over the culture period (*p* ≤ 0.0001). The release of PDGF-BB indicates the cells continued to recruit perivascular cells in order to maintain structural integrity. For the co-culture model, a significantly lower level of PDGF-BB was produced after 3 days of post-HUVECs seeding as compared to the HUVECs alone group (*p* ≤ 0.0001). Once the hMSCs were added at day 3 to the pre-seeded cellular scaffolds, the levels of PDGF-BB were diminished and no longer detected, where no significant differences were observed at a later time-point (*p* ≥ 0.05). Concurrently, the co-culture model produced significant levels of VEGF over the culture period (*p* ≤ 0.0001). hMSCs monoculture showed no significant differences in PDGF-BB secretion over time (*p* ≥ 0.05).

In contrast, the hMSCs alone group released a substantially high level of VEGF over the culture period. There is an increment in the level of VEGF secreted by the hMSCs alone from day 3 to day 10 (*p* ≤ 0.0001). However, HUVECs alone showed almost negligible levels of VEGF production, indicating nearly no protein was released in this monoculture. The co-culture model demonstrated similar trends to those of the hMSC monoculture. Before the addition of hMSCs, post-HUVECs seeded scaffolds produced a negligible level of VEGF as the HUVECs alone (*p* ≥ 0.05). The levels of VEGF increased drastically with the addition of hMSCs after 3 days post-HUVECs seeding. The co-culture system continues to secrete more VEGF over the culture period.

## 4. Discussion

Various 3D scaffolds have been utilised in culturing stem cells in scaffold-based tissue engineering. When introduced into these scaffolds, provided with optimal culture conditions and chemical cues, the cells can undergo proliferation, differentiation, and secretion of specific extracellular matrix (ECM) molecules [19,23]. When it comes to bone tissue engineering (BTE), scaffolds should meet specific criteria to be effective. It must have a 3D structure, sufficient porosity with an open pore network, biocompatible, and biodegradable. These characteristics are crucial to creating a scaffold that can support successful BTE applications. Among the fabrication methods, 3D printing (3DP) is considered valuable for fabricating BTE scaffolds, particularly by fused deposition modelling (FDM), which uses biodegradable and biocompatible polyesters such as poly (lactic) acid (PLA) or polycaprolactone (PCL). Surface modification by introducing ceramics such as hydroxyapatite (HA), calcium silicate (CaSi), and bioglass (BG) on these scaffolds has been reported to improve osteogenesis.

Previous research has shown several post-fabrication modifications of the polymeric scaffolds with bioceramics. Recently, the potential of 3DP PCL scaffolds coated with hydroxyapatite (HA) and bioglass (BG) has been reported. These findings demonstrated that the synergistic effect of HA and BG resulted in higher in vitro cell viability and bone formation as compared to PCL, PCL/HA, and PCL/BG scaffolds [3]. In our previous study, we successfully developed a novel polyelectrolyte multilayers coating consisting of 5-bilayers silicon carbonated hydroxyapatite nanopowders dispersed in collagen type I and hyaluronic acid on the 2D PLA films [12]. This optimal condition was then adapted to fabricating 3DP hybrid scaffolds. This study aimed to correlate the fabricated scaffolds’ structural design in determining stem cells’ fate when cultured in different culture media under static and dynamic culture conditions. The vascular network within matured bone could be likened to channels through which nutrients can be supplied to cells and tissues. In order to re-create this vascular network, millimetre-sized channels (diameter = 1.5 mm) were created on the 2C and 4C scaffolds. These channels were designed to facilitate the dispersion of hMSCs throughout the scaffolds and encourage nutrient exchange to promote differentiation, particularly under dynamic culture conditions.

A rotating wall vessel (RWV), also known as a rotary bioreactor, is known to create the effect of microgravity simulation. This type of bioreactor was used to minimise shear force and maximise the fluid flow throughout the scaffolds to provide enhanced mass transfer across large scaffolds for BTE [18,30]. Pure sintered HA scaffolds were used as the experimental controls. The HA scaffold is commercially available and has been investigated for its clinical relevance. Thus, it was assumed as the “golden standard” in this study. The in-house fabricated 3DP hybrid scaffolds were compared to the pure HA scaffolds in order to select the best scaffold that serves as the optimum structural and functional design for potential BTE applications.

The live/dead stain indicated that 3DP hybrid scaffolds allow greater cell attachment than pure HA scaffolds during seeding. It takes only 24 h for the cells to properly attach to the former scaffolds, while the later scaffold requires at least 72 h. Collagen is an adhesion protein, favouring cell attachment and proliferation [31,32]. Also, the literature has reported that culturing bone-marrow stromal cells (BMSCs) on HA/Collagen coated PCL scaffolds revealed more viable cells and higher ALP activities than those in collagen-coated alone or uncoated PCL scaffolds [33]. Thus, the presence of SiCHA ceramics in collagen matrix on 3DP hybrid scaffolds allowed easier and faster cell attachment compared to pure HA scaffolds.

Cells seeded on HA scaffolds survived only in static conditions and were found to be completely detached once cultured in dynamic conditions. Culturing 3DP hybrid scaffolds in OM and PM shows notable differences, particularly under microgravity simulation. For instance, after 21 days in OM, cells started forming aggregates/bone-like nodules on 4C cellular scaffolds, which indicates the early sign of osteogenic differentiation.

Quantification of DNA for experimental groups demonstrated higher levels of DNA were obtained when all scaffolds were cultured under static conditions in PM over time, which indicated that the cells were actively proliferating, increasing cell numbers, and therefore, increasing the amount of DNA. However, when exposed to microgravity in PM, lower DNA contents were obtained compared to other culture conditions. Without osteogenic supplements, proliferation media can only help in cell expansion to achieve into higher cell number [34,35]. Thus, as the culture progressed, these scaffolds with greater cell density than the surrounding medium started to sediment to the side of the vessel and experienced repeated friction with the vessel wall. As a result, some cells detached from the scaffolds and were floating in the culture media. For instance, when HA scaffolds were cultured in dynamic compared to static condition, the majority of the cells had detached from the scaffolds at an early stage of culture, thus resulting in a negligible amount of DNA detected at day 21. hMSCs are known to be anchorage-dependent cells; thus, they need a substrate to attach and survive in culture [34]. It is assumed that when the hMSCs were in a suspension state, the detached cells were floating in the continuously rotating culture media, which eventually cause cell death. This explained why we obtained lower DNA contents and a higher percentage of LDH activity for scaffolds cultured under dynamic flow in PM. In contrast, cells in osteogenic media for both culture conditions seem to survive better than those in PM. The presence of dexamethasone, β-glycerolphosphate, and ascorbic acid in OM has driven the cells towards osteoblastic differentiation over time [36,37]. It is believed that better cell adhesion was provided since more ECM is produced as the cells undergo differentiation. As a result, fewer cells detached from the scaffolds cultured in Dynamic/OM compared to Dynamic/PM, which was represented by the low %LDH activity.

A lactate dehydrogenase (LDH) assay was performed to quantify the percentage of cytotoxicity in the culture medium, predicting the phenomena happening in Dynamic/PM culture condition. Lactate is mainly produced from glucose metabolism. Glutamine can also excrete a small amount of lactate. The lactate concentration depends on the glucose concentration, cellular activity, and bioreactor operation. Higher shear induced by the bioreactor resulted in higher lactate concentrations in the culture medium. The presence of lactate is then likely to impede cell growth and metabolism and decrease productivity [38]. This is due to the changes in the osmolarity of the media where lactate is attributed to media acidification. Consequently, growth may be restricted by lactate even at constant pH. This phenomenon resulted in the cellular scaffolds’ down-regulation of cell activity and total protein production in Dynamic/PM culture condition.

Contrarily, more proteinaceous materials were produced when hMSCs started differentiating into the osteogenic lineage. This is observed when the cellular scaffolds were cultured in OM and, more apparent, in Dynamic/OM. It has been reported that an early stage of osteogenic differentiation is the expression of collagen type I matrix onto which the mineral is deposited. While in the final stage, from day 14 to 28, high expression of osteocalcin and osteopontin is usually obtained. This is followed by the deposition of calcium and phosphate. However, in this work, the total protein produced by the cellular scaffolds was not analysed in detail to classify the different types of bone-synthesised proteins.

hMSCs cultured on 3DP hybrid scaffolds in OM exhibited higher ALP activity than those in PM despite the culture condition used. 4C cellular scaffolds in osteogenic media for both culture conditions showed the highest levels of ALP activity after 21 days. This indicates that the 4C scaffold is the most favourable substrate for hMSCs cultured in microgravity simulation, as cells were able to proliferate and differentiate the fastest in this condition. On the other hand, HA scaffolds showed almost negligible amount of ALP expression when cultured under microgravity simulation in both culture media. This is due to the cell detachment from the scaffolds at the early stage of culture. As the culture progressed, hMSCs started to differentiate into the osteogenic lineage, with cells becoming alkaline phosphatase (ALP)-positive histochemically, in particular for the 3DP hybrid scaffolds under the dynamic condition in OM after 21 days of culture, i.e., 4C in Dynamic/OM. ALP histochemical analysis is considered one of the earliest phenotypic markers of the osteoblastic lineage, indicating the mineralisation onset.

μ-CT analysis effectively monitors the mineralised matrix formation within 3D tissue-engineered constructs in vitro and in vivo. In this study, μ-CT analysis was used to detect further if any mineralisation formed on the cellular scaffolds after exposure to different culture conditions. μ-CT analysis revealed the most coverage with a denser area on the 4C cellular scaffolds in OM after 21 days compared to other investigated scaffolds. Correlating these results with the formation of bone-like nodules observed in live/dead staining and positive ALP expression, these denser areas can be assumed as mineralised matrix formation. A denser mineralised layer at the surface of cellular constructs is a common observation in tissue engineering [39]. Quantitative μ-CT analysis supported this observation, where 4C scaffolds in OM exhibited huge increments in the total volume and highest percentages of mineralisation compared to other scaffold designs in different culture conditions.

Our finding highlights that the in-house fabricated 3DP hybrid scaffolds performed better compared to the pure HA in all culture conditions. HA alone is insufficient to enhance cell attachment and induce osteogenic differentiation, particularly in dynamic conditions, even with the help of biochemical cues from the osteogenic media. Native bone tissue comprises two core components: the first is the mineralised inorganic phase consisting mainly of calcium phosphate with multiple ionic substitutions, and the second is the non-mineralized organic phase, which is predominantly collagen type I. Therefore, natural bone is more accurately referred to as carbonated hydroxyapatite as carbonate ions are the most abundant rather than solely hydroxyapatite [40]. It is believed that there was a lack of cell recognition when cells were cultured on HA scaffolds, although they are known to be osteoconductive materials. This might also explain the weak bonding between cells and HA scaffolds in a dynamic culture. The 3DP hybrid scaffolds were built from a combination of both osteoconductive and osteoinductive materials, with multi-substituted HA and collagen type I as the major components of the coating materials. These scaffolds closely resemble the compositions of natural bone. Thus, they are more likely to behave similarly to the bone compared to monolithic scaffolds. In addition, the presence of multi-substituted HA (SiCHA) powders has influenced the cellular responses of the hMSCs on the 3DP hybrid scaffolds. It is known that multi-substituted HA powders can provide better bioactivity by raising their solubility compared to pure HA.

This study demonstrated the relationship between scaffold pore size and cell activity within tissue engineering constructs. As shown on HA scaffolds, smaller pore sizes prevented cellular penetration and extracellular matrix production [4]. Larger pore sizes were seen to improve the overall performance of the hMSCs cultured on 3DP hybrid scaffolds, particularly in dynamic environments. It is believed that the presence of larger pores might have allowed for homogenous fluid flow in the bioreactor, hence minimising shear and turbulences around the scaffold peripheries, which facilitate cell penetration and migration throughout the entire scaffolds. Among the 3DP hybrid scaffolds, 4C scaffolds were considered the best scaffold design under the here investigated culture conditions. Indeed, mesh scaffolds possess a higher surface area as compared to 2C and 4C scaffolds since they have the highest porosity, which is important for cell penetration. However, cells are subjected to excessive fluid shear, particularly when they are exposed to a dynamic culture. As a result, cells that adhered to the struts of the mesh scaffolds were washed off. In this case, channel scaffolds, which have a relatively smaller SA:V ratio, offered some shelter for the cells from excessive fluid shear and, at the same time, still permitted suitable nutrient flow. At this stage of the study, it can be concluded that 4C scaffolds are the most promising scaffold designs as compared to 2C and mesh scaffolds. This is because 4C scaffolds possess suitable surface area for cell attachment and porosity to allow mass transfer than 2C and mesh scaffolds.

## 5. Conclusions

The structural design of the 3DP hybrid scaffolds has a pronounced impact on the behaviour of hMSCs in vitro. In addition, the combination of dynamic culture based on microgravity simulation and different culture media also plays an essential role in determining cell fate. Our in-house fabricated 3DP hybrid scaffolds as cell culture substrates enhanced cell proliferation and differentiation compared to the control scaffold in all culture conditions. The 4C hybrid scaffolds showed the best performance among the scaffold designs investigated. It is concluded that creating millimetre-size aligned channels on the scaffold structure to resemble a vascular structure has greatly facilitated cell migration, proliferation, and differentiation under the dynamic environment in osteogenic media. The concept of initiating blood vessel formation in 4C hybrid scaffolds has also demonstrated successful growth and multiplication of Human Umbilical Vein Endothelial Cells (HUVECs) and human Mesenchymal Stem Cells (hMSCs) within the structure. Manufacturing these in-house 3DP hybrid scaffolds with multilayered ECM-like coating provides a new dimension in bone regeneration.

## Figures and Tables

**Table 1:** The total volume of the dry scaffolds (before seeding) and the cellular scaffolds under different culture conditions after 21 days (mean ± standard deviation; *n* = 3).

Condition/ Scaffold	Volume (mm^3^)			
	HA	2C	4C	MESH
**Dry**	35.27 ± 0.55	80.20 ± 0.32	81.90 ± 0.28	80.77 ± 0.35
**Static/OM**	38.71 ± 0.48	90.30 ± 0.25	93.72 ± 0.22	86.67 ± 0.31
**Static/PM**	37.38 ± 0.50	82.91 ± 0.21	85.06 ± 0.15	83.49 ± 0.23
**Dynamic/OM**	36.18 ± 1.51	88.85 ± 0.30	95.41 ± 0.22	96.13 ± 0.52
**Dynamic/PM**	35.49 ± 1.39	82.14 ± 0.45	83.89 ± 0.39	82.36 ± 0.49

**Table 2:** The percentage of mineralisation of the cellular scaffolds under different culture conditions after 21 days (mean ± standard deviation; *n* = 3).

Condition/ Scaffold	Mineralisation (%)			
	HA	2C	4C	MESH
**Dry**	-	-	-	-
**Static/OM**	2.07 ± 0.13	12.06 ± 0.32	22.64 ± 0.19	11.65 ± 0.38
**Static/PM**	2.15 ± 0.22	8.43 ± 0.38	8.59 ± 0.17	7.98 ± 0.23
**Dynamic/OM**	0.53 ± 0.18	12.31 ± 0.35	26.94 ± 0.11	16.38 ± 0.41
**Dynamic/PM**	0.39 ± 0.10	4.63 ± 0.27	5.15 ± 0.10	4.81 ± 0.19

## Data Availability

Data are contained within the article.
